# A Treatise on the Medical Jurisprudence of Insanity

**Published:** 1839-04-01

**Authors:** 


### A Treatise on the Medical Jurisprudence of Insanity. By *J. Ray*, M.D. Boston, 1838.

The very imperfect state of our medical literature with respect to works on insanity, considered exclusively in its legal relations, is acknowledged on all hands. Whilst so much has been done in promoting the comfort and ameliorating the treatment of the insane, it is truly melancholy to reflect how little has been accomplished towards regulating their personal and social rights, by more correct principles of legislation. In every thing connected with the legal relations of this unfortunate class of persons, we seem disposed to remain content with, and to go on rejoicing in, the wisdom of our ancestors. Some share of blame for this must perhaps attach to medical men, who have not bestirred themselves to obtain, for the results of their researches, that influence on the jurisprudence of insanity, which they have been so successful in obtaining over its pathology and treatment. In proof of the existence of this apathy we may mention, that the English language does not contain a work in which the various forms and degrees of mental derangement are treated, in reference to their effect on the rights and duties of man, if we except Dr. Haslam's work, which, however, is far too brief as well as too general, to be of any practical utility as a book of reference. Germany has had the start of us in this, as well as in every other branch of forensic medicine, Dr. Hoffbauer having published a treatise on insanity in connexion with its legal relations, of very great merit. In France too, Dr.

Georget has cultivated this field of enquiry with great zeal and success, and has produced a very excellent little work, entitled "Maladies Mentales, considerées dans leurs Rapports avec la Legislation Civile et Criminelle." When it is considered that opinions on insanity viewed in the light of a disease—of a derangement of the physical structure—have been for some time back progressively changing for the better; it will be readily acknowledged that its legal relations, which should obviously be determined in some measure at least by our views of its nature, ought to be modified by the progress of our knowledge. A work therefore on this subject, to be of real practical utility, must come from a practical physician, as a mere legal practitioner, who will consider insanity less in its pathological than its psychological relations, will be disposed to attach too little importance to its connexion with physical causes.

We have every day proofs in our courts of justice, that the principles laid down on the subject of insanity by legal authorities have received too much of that respect and veneration which is naturally felt for the opinions and practices of our ancestors, and that all innovations or attempts at improvement have been too much regarded rather as the offspring of new-fangled theories, than of the steady advance of medical science. Hence it is that the principles of legislation, in reference to insanity, have not kept pace with our knowledge of the nature and treatment of this disease.

Various divisions of the insane have been proposed by legislators and jurists. Lord Coke divides those *non compotes mentis* into four classes:—1, an idiot, who from his nativity by a perpetual infirmity is *non compos*; 2, he that by sickness, grief or other accident, wholly loseth his memory and understanding; 3, a lunatic that hath sometimes his understanding, and sometimes not; 4, he that by his own vicious act for a time depriveth himself of his memory and understanding, as he that is drunken.

The defect of this division appears at once from the difficulty experienced by law-writers to point out the precise characters by which each class may be distinguished. "An idiot is defined to be a person, who cannot count or number twenty pence, or tell who was his father or mother, or how old he is, so as it may appear that he hath no understanding of reason, what shall be for his profit, or what shall be for his loss; but if he have sufficient understanding to know and understand his letters, and to read by teaching or information, he is not an idiot. Now the truth is, that the proportion of idiots, capable of attaining the kind of knowledge herein specified, by means of the ordinary intercourse with men, or of special teaching, is by no means small. The entire loss of memory attributed to the second class, is observed only as a sequel to madness or some other disease, or as the result of some powerful moral causes; so that if this is to be considered an essential character of madness, by much the larger proportion of madmen will be altogether excluded from this classification." "Judging from the almost exclusive use of the term *lunacy*, and the frequent reference to lucid intervals, the intermittent character of madness was either more common, some hundred of years since, or, which is more probable, in consequence of the general belief in its connexion with lunar influences, this intermission was imagined to occur far oftener than it really did."

Originally, commissions of lunacy were granted for the purpose of enquiring whether the individual was either an idiot *ex nativitate*, or a lunatic.

The obvious inadequacy of the division of the insane into idiots and lunatics, and the gross injustice of leaving out of the protection of law that larger class of insane, who, though neither *idiots*, nor *lunatics*, labour under more or less mental derangement, led to a change in the form of the writ, by which the phrase *unsound mind* was used for the purpose of embracing all others, who were considered proper objects of a commission.

Here again another difficulty presented itself: no fixed meaning was attached to the term *unsound mind*. From the feelings of disgust with which madness has generally been regarded, juries have frequently been unwilling to return a verdict of unsound mind, which in the popular acceptation was equivalent to insanity, though the individual's mental faculties may be so enfeebled as to render him totally unequal to the management of his affairs. And when the jury have returned the verdict of incapacity from weakness of intellect, the commission was quashed for want of the accompanying qualification of unsoundness of mind. The absurdity of this pertinaceous adherence to technical phraseology must certainly strike every impartial mind.

"The object of the commission is, to ascertain whether or not the party in question is incapable, by reason of mental infirmities, of governing himself and managing his affairs; and if they so find him, it certainly is irrelevant to any useful purpose, to connect this inability as an effect with any particular kind of insanity, whether expressed in common or technical language. Indeed to require a jury to infer explicitly unsoundness of mind from inability to manage affairs, which is of itself sufficient evidence of all the mental unsoundness, that is required for practical purposes, and reject their return if they do not, would seem exceedingly puerile, were it not strictly professional."

Another instance, where the principles of common sense and common justice, which ought to regulate the legal relations of the insane, have been strangely disregarded in the maxims of the common law, is this: it requires that contracts to be valid, should spring from a free and deliberate consent, and yet it refuses to suffer the party himself to avoid them on the plea of lunacy, in accordance with an antient maxim, that no man of full age shall be allowed to disable or stultify himself; though, at the same time, it does allow his heirs, or other persons interested, to avail themselves of this privilege. Thus a person who recovers from a temporary insanity, before the return of an inquisition, has no remedy in law or in equity for the most ruinous contracts that he may have entered into while in that condition, except on the ground of fraud, though, after his death, his heirs may have them set aside by establishing the fact of lunacy alone.

Nearly the same respect for antiquated maxims is entertained in the application of the law to criminal cases. Lord Hale, in laying down the law in respect to criminal acts, divides insanity into two species, the partial and total; the former of which is not allowed to exempt from responsibility for crime. This distinction has been on some occasions acted on in our criminal courts. In the case of Bellingham, who shot Mr. Percival, the then Attorney-General said, "a man may be deranged in his mind—his intellects may be insufficient for enabling him to conduct the common affairs of life, such as disposing of his property, or judging of the claims which his respective relations have upon him, and if he be so, the administration of the country will take his affairs into their management, and appoint to him trustees; but at the same time such a man is not discharged from his responsibility for criminal acts."

Lord Erskine had previously given the same doctrine the sanction of his authority in his celebrated speech in defence of Hadfield: "I am bound," he says, "to admit, that there is a wide distinction between civil and criminal cases. If, in the former, a man appears upon the evidence, to be *non compos mentis*, the law avoids his act, though it cannot be traced or connected with the morbid imagination which constitutes his disease, and which may be extremely partial in its influence upon conduct; but to deliver a man from responsibility from crimes, but above all from crimes of great atrocity and wickedness, I am by no means prepared to apply this rule, however well established, when property only is concerned."

That a person prevented by law from managing his own affairs, in consequence of impaired intellect, should, in respect to criminal acts, be held responsible, is a proposition so startling, that it is impossible to look on it in any other light than as belonging to that class of doctrines "which, while they may be the perfection of reason to the initiated, appear to be the height of absurdity to every one else."

Our author's animadversions on this piece of law, are so much in point, that we cannot refrain from giving them in his own words. "The language of the law virtually addressed to the insane man is, your reason is too much impaired to manage your property; you are unable to distinguish between those measures which would conduce to your profit, and such as would end in your ruin; and therefore it is wisely taken altogether from your control; but if, under the influence of one of those insane delusions, that have rendered this step necessary, you should kill your neighbour, you will be supposed to have acted under the guidance of a sound reason; you will be tried, convicted, and executed like any common criminal, whose understanding has never been touched by madness."

Another distinction made between civil and criminal cases, regards the evidence respecting the state of the party's mind. In the former, proof drawn from the nature of the act is paramount to all others, and, in the absence of others, is admitted to be conclusive; whilst in the latter, an attempt to prove insanity from the character of the act, would be looked on as a begging of the question, a regular running round in a circle.

In the trial of Hadfield, for shooting at the King in Drury-lane Theatre, the Attorney-general had told the jury that to exempt a person from criminal responsibility there must be a total deprivation of memory and understanding. To this Mr. Erskine, counsel for Hadfield, replied that, if the terms were to be understood in the literal sense, no such madness ever existed. He was the first lawyer, in an English court of justice, who laid it down that the true character of insanity, which would exempt from responsibility, was *delusion*, of which the criminal act in question must be the immediate unqualified offspring.

A doctrine laid down more than once, on the trials of insane persons for homicide, was, that if a person were capable, in other respects, of distinguishing right from wrong, there was no excuse for any act of atrocity, which he might commit under this description of derangement. Now, as the author well observes, the insane mind is not entirely deprived of the faculty of distinguishing right from wrong, but on many subjects it is perfectly rational, as is very well known.

"The first result, therefore, to which the doctrine leads, is, that no man can ever successfully plead insanity in defence of crime, because it can be said of no one, who would have occasion for such a defence, that he was unable in any case to distinguish right from wrong. To show the full merits of the question, however, it is necessary to examine more particularly, how far this moral sentiment is affected by, and what relation it bears to insanity. By that partial possession of the reasoning powers, which has been spoken of as enjoyed by maniacs generally, is meant to be implied the undiminished power of the mind, to contemplate some objects or ideas in their customary relations, among which are those pertaining to their right or wrong, their good or evil, tendency; and it must comprise the whole of these relations, else the individual is not sane on these points. A person may regard his child with the feelings natural to the paternal bosom, at the very moment he believes himself commanded by a voice from Heaven to sacrifice this child, in order to secure its eternal happiness, than which, of course, he could not accomplish a greater good. The conviction of a maniac's soundness, on certain subjects, is based in part on the moral aspect in which he views those subjects; for it would be folly to consider a person rational, in reference to his parents and children, while he labours under an idea that it would be doing God's service to kill them; though he may talk rationally of their characters, dispositions, and habits of life, their chances of success in their occupations, their past circumstances, and of the feelings of affection which he has always cherished towards them.

Before, therefore, an individual can be accounted sane on a particular subject, it must appear that he regards it correctly, in all its relations to right and wrong. The slightest acquaintance with the insane, will convince any one of the truth of this position. In no school of logic, in no assembly of the just, can we listen to closer and shrewder argumentation, to warmer exhortations to duty, to more glowing descriptions of the beauty of virtue, or more indignant denunciations of evil doing, than in the hospitals and asylums for the insane. And yet many of these very people may make no secret of entertaining notions utterly subversive of all moral propriety; and, perhaps, are only waiting a favorable opportunity to execute some project of wild and cruel violence. The purest minds cannot express greater horror and loathing of various crimes than madmen often do, and from precisely the same causes. Their abstract conceptions of crime, not being perverted by the influence of disease, present its hideous outlines as strongly defined as they ever were in the healthiest condition; and the disapprobation they express at the sight, arises from sincere and honest convictions. The *particular* criminal act, however, becomes divorced in their minds from its relations to crime in the *abstract;* and, being regarded only in connexion with some favorite object, which it may help to obtain, and which they see no reason to refrain from pursuing, is viewed, in fact, as of a highly laudable and meritorious nature. Herein, then, consists their insanity; not in preferring vice to virtue, in applauding crime and ridiculing justice, but in being unable to discern the essential identity of nature, between a particular crime and all other crimes, whereby they are led to approve what, in general terms, they have already condemned."

We have cited this passage at full length, in the author's own words, as we consider that the absurdity of making the moral discernment of right and wrong in the abstract, a test of sane mind, could not be exhibited more clearly or more satisfactorily.

Another criterion of responsibility in doubtful cases, is the design manifested in the commission of the criminal act. Those who see a disproof of the existence of insanity in the possession of systematic design, must have made but little progress in the study of madness; any one acquainted with the inmates of lunatic asylums, must have often heard of the ingenuity of contrivance and adroitness of execution, which characterize the plans of the insane.

Having now demonstrated the fallacious nature of the two tests of responsibility just mentioned, namely, the power of discriminating right and wrong, and the power of design, the author comes to the consideration of the test proposed by Mr. Erskine, scil. *delusion;* the correctness and sufficiency of which, he says, would be unobjectionable, if our intellectual faculties alone were liable to derangement.

"But it must not be forgotten," he says, "that the Author of our being has also endowed us with certain moral faculties, comprising the various sentiments, propensities and affections, which, like the intellect, being connected with the brain, are necessarily affected by pathological actions in that organism. The abnormal condition thus produced, may exert an astonishing influence on the conduct, changing the peaceable and retiring individual into a demon of fury, or, at the least, turning him from the calm and quiet of his lawful and innocent occupations, into a career of shameless dissipation and debauchery, while the intellectual perceptions seem to have lost none of their ordinary soundness and vigor."

All this evidently involves the doctrine of moral insanity; a doctrine, by the way, which has been received rather unfavorably by judicial authorities, not certainly for want of sufficient facts to support it, but from the tendency of the mind to resist all innovations, which may at all come in collision with old and generally received prejudices. When this doctrine was advanced some time since in behalf of a person on trial for murder, it was denounced by the court as a "groundless theory."

"Such opinions," observes the author, "from quarters where a modest teachableness would have been more becoming than an arrogant contempt for the results of other men's inquiries, involuntarily suggest to the mind a comparison of their authors with the saintly persecutors of Galileo, who resolved, by solemn statutes, that nature always had operated, and always should operate, in accordance with their views of propriety and truth."

The test of "delusion" then, in cases of doubtful insanity, is not much better than those already mentioned; nor is there any single, isolated character, which may not be equally objected to. Insanity is a disease, and, as with all other diseases, the fact of its existence is never established by a single diagnostic symptom, but by the whole body of symptoms. It is readily admitted by every one that the faculty of distinguishing the manifestations of health from those of disease requires tact, judgment, and experience; every one allows this in reference to the lungs, heart, liver, etc. Why not admit the same with respect to the brain? and much more readily too, in as much as from the very obscure nature, and the ever varying phenomena of its functions, more tact, more knowledge is required, and consequently more difficulty is experienced in appreciating its several manifestations both in health and disease, than in the case of the other organs of the body. The lawyer, perhaps, will say that in arguing thus, we are setting up a barrier between the domain of professional knowledge and that of common sense; to which remark a very simple reply at once suggests itself; viz.

that when the lawyer is himself attacked, or has any friend for whom he is deeply interested attacked, with pulmonary, hepatic, or any other disease, he does not feel himself warranted in appealing to the common sense of some professional brother for the purpose of having the nature of his disease determined, and the proper treatment pointed out. What then does he do? He calls in the physician, who has attained professional reputation for skill, tact, and experience in treating the particular disease in question. What, we would say, is this, that facts, established by men of undoubted experience and good faith, should not be rejected as mere "groundless theory," because they happen to be novel, and to come in collision with the antiquated prejudices of professional routine, taken up without examination, and adhered to solely, because our predecessors acted on them. Common justice, common humanity, and the remonstrances of modern science will, however, no longer tolerate the employment of the argumentum ad verecundiam, that hackneyed and now justly despised weapon of obstinate bigotry in cases where the interests of so large a portion of our fellow-creatures are so deeply involved. That there are cases where we are compelled to rest satisfied with the argument of authority, we are free to admit; we deny, however, that the subject of insanity is one of these cases; knowledge on this subject is not at a standstill; our acquaintance with the nature, pathology, and treatment of this disease, is daily advancing; it is therefore but reasonable to expect that the legislative enactments regarding it, which should be made to conform to the nature of the disease, should also undergo those modifications suggested by the improvements in our knowledge of it.

It has been said that "all crime is the result of partial insanity;" and the inference intended to be drawn was, that partial insanity is no excuse for guilt. Here we have a precious instance of the confusion introduced into reasoning, when vague and ambiguous terms are employed; this frequently happens when the positive act, and its moral relation are designated by the same term, as here the term *crime* is obviously used to signify both the positive act of the individual, and the relation of that act to an established standard or law. Mr. Locke adduces the term *stealing*, if we forget not, in illustration of a similar confusion arising from ambiguity of terms, in his chapter on Moral Relations. The judge or jurist, who, for the purpose of establishing the inference, that insanity does not exempt from responsibility, lays it down that crime is the result of insanity, is himself involved in a glaring petitio principii by his using the term "*crime*," which in popular and general usage includes in it the idea of guilt.

But crime, in the strict and proper acceptation of the term, is not necessarily the result of insanity, even when perpetrated under the excitement of the most violent passions. Crime, to be crime, is always suggested by motives, insufficient it may be, but still rational motives, having reference to definite and real objects.

Violent passions may impair the judgment, but they do not vitiate the perceptions, nor deprive the mind of its power of comparison. But the case is very different in mental derangement.

"The causes which urge the insane to deeds of violence, are generally illusory—the hallucinations of a diseased brain—or they may act from no motive at all, solely in obedience to a blind impulse, with no end to obtain, nor wish to gratify. Madness, too, is more or less independent of the exciting causes, that have given rise to it, and exists long after those causes have been removed, and after the paramount wish or object has been obtained. In short, madness is the result of a certain pathological condition of the brain, while the criminal effects of violent passions merely indicate unusual strength of those passions, or a deficient education of those higher and nobler faculties that furnish the necessary restraint upon their power. It is admitted that strong passions do deprive the individual of the power of calmly deliberating and perceiving the terrible consequences of his fury; and legislators have wisely distinguished it from deliberate, premeditated mischief, by uniting it with a minor degree of punishment. In drunkenness the same effect is produced to such a degree as to amount to temporary insanity; but neither does this, any more than strong passions, exempt from all punishment; for the plain reason, that, in each case, the impairment of moral liberty is the voluntary act of the individual himself."

According to the French penal code, if the existence of insanity is once established, the responsibility of the party is at once taken away. As it is not quite certain, however, that insanity under every form or circumstance whatever, ought to annul a person's criminal or civil liabilities, and moreover as allowing the court or jury any latitude on this point, would just be equivalent to having no law whatever on the subject, our author suggests that the mental unsoundness, in order to exempt from responsibility, should be required by the law to have embraced the criminal act within its influence. This would narrow the question for the jury and leave them merely the fact of insanity to determine.

As the decision of the jury, relative to the existence of insanity, must be based on the testimony of the witnesses adduced, it becomes a matter of great importance by whom this testimony is given. If the business of the witnesses on such occasions was to testify to a simple matter of fact, it might perhaps be of little consequence who these witnesses may be; but when we know that the chief business of such witnesses is to deliver opinions, which are to shape the final decision, there should, as the author observes, be some qualification required on the part of those who give such opinions, not required of one who testifies to mere facts. If a particular class of men only are thought capable of treating insanity, it very naturally follows, as a matter of course, that such only are capable of giving opinions on judicial proceedings relative to insanity. To remedy the difficulties arising from the contradictory opinions so often given by medical men on trials relative to insanity, by which the minds of the jurors are so perplexed and confounded, the author proposes to appoint a class of men somewhat like the *experts* of the French, peculiarly fitted for the duty, by a course of studies expressly directed to this end.

To account for the little progress, comparatively speaking, made by medical men in the knowledge of insanity, some persons allege that the study of mental philosophy, or that of mind in the healthy state, which is necessary in order to have correct notions in its disordered condition, has been too much neglected. The author who, we strongly suspect, is an ardent disciple of Gall, thinks on the contrary, that the result in question has been owing to the undue account that physicians have made of the popular philosophy of mind in explaining the phenomena of insanity, and that they have failed in consequence of studying metaphysics too much instead of too little. He conceives that the science of metaphysics, as we now have it, is utterly incompetent to furnish a satisfactory explanation of the phenomena of insanity and that a more deplorable waste of ingenuity can scarcely be imagined, than that evinced in the modern attempts made to reconcile the facts of the one with the speculations of the other. In proof of his assertion he instances the case of monomania, the existence of which, as a distinct form of mental derangement, was denied, and considered to be a mere fiction of medical men, long after it had been admitted among the established truths of science. How far the author may be correct in these, his views, regarding the detrimental influence of metaphysics, and the paramount utility of phrenology, we shall not stop to consider.

The author having thus far presented the reader with some general views regarding the legal relations of the insane, and the nature and extent of their responsibility in civil and criminal matters, having pointed out also what he conceives to be the cause of the little progress hitherto made in our knowledge of the nature and treatment of mental derangement, now proceeds to enter into a more systematic and detailed account of his subject; he first commences with the consideration of :—

### Mental Diseases in General.

He assumes as an undoubted truth, what very few will at the present day be found to deny, that the manifestations of the intellect, and those of the sentiments, propensities, and passions, or generally, of the intellectual and affective powers, are connected with, and dependent on the brain; he then infers that abnormal conditions of these powers are equally connected with abnormal conditions of the brain. The truth of this inference has been actually established by post-mortem examinations, deviations from healthy structure being generally found in the brains of insane subjects. A natural classification of the various forms of insanity, though of secondary importance in regard to its medical treatment, must prove of eminent service to the legal enquirer, by enlarging his notions of its phenomena, and enabling him to discriminate, where discrimination is necessary to the attainment of important ends. The deplorable consequences of knowing but one kind of insanity, and of erecting that into a standard, whereby every other is to be compared and tested, are too common in the records of criminal jurisprudence; and it is time, observes the author, that it were well understood, that the philosophy of such a method is no better than would be that of the physician who should recognize no diseases of the stomach for instance, but such as proceed from inflammation, and reject all others as anomalous or unworthy of attention. The various diseases included under insanity admit of being divided under two heads, founded on two very different conditions of the brain; the first being a want of ordinary development, the second some lesion of structure subsequent to its development. Under the former head he places idiotcy and imbecility, under the latter, mania and dementia. He adopts, in fact, Esquirol's division, for a full analysis of which excellent work we beg to refer the reader to some of the preceding numbers of this Journal; we shall therefore proceed to that part of the work where the author lays down "the legal consequences of mental deficiency."

"Our moral and our intellectual constitution is constructed in harmony with the external world on which it acts, and by which it is acted upon ; the result of this mutual action being the happiness and spiritual advancement of an immortal being. Thus endowed with the powers of performing the part allotted us, and placed in a situation suitable for exercising and developing them, we become accountable for the manner in which they are used. All legal responsibility, therefore, is founded on this principle of adaptation, and ceases whenever either of its elements is taken away. The intellect must not only be sufficiently developed, to acquaint the individual with the existence of external objects, and with some of their relations to him, but the moral powers must be sound enough and strong enough to furnish, each its specific incentives, to pursue that course of conduct, which the intellect has already pointed out. It is nothing that the mind is competent to discern some of the most ordinary relations of things, and is sensible of the impropriety of certain actions; so long as the individual is incapable, by defect of contitution, of feeling the influence of those hopes and fears, and of all those sentiments and affections that man naturally possesses, an essential element of legal responsibility is wanting, and he is not fully accountable for his actions."

The author first considers the case of imbeciles; he shows the absurdity of making such persons responsible for their actions in the same degree as one enjoying the full vigour and soundness of the higher faculties; because an essential element of responsibility is a power to refrain from evil doing, which power is furnished by the exercise of those faculties, which are but imperfectly, if at all, developed in the imbecile. The acts of the imbecile, for want of the nobler faculties, are contemplated by him solely in relation to himself; he never for a moment considers the consequences of such acts in relation to others, It is no easy matter to satisfy many persons, however, that an individual who labours under no delusion, and who enjoys a certain degree at least of moral liberty, should not be held responsible for his acts. This error arises from the vulgar habit of estimating the strength and extent of the moral faculties by the ability to go through certain mechanical duties with tolerable success.

The author here cites several cases in illustration of his views on the nature of imbecility, which want of space obliges us to pass over.

On the subject of moral mania, and the responsibilities of those labouring under that form of insanity, our author has been peculiarly felicitous. Moral mania is a matter looked on as visionary even at the present day in criminal courts, when urged in defence of the insane. It has been too much the custom to limit the influence of insanity solely to the intellectual faculties. Surely no one will deny that the propensities and sentiments are as much integral portions of our mental constitution as the intellectual faculties, and that their manifestations are just as much dependent on the cerebral organization. To Pinel the world is indebted for first directing attention to the existence of moral mania, as a distinct form of disease. Before his time, it was the universal belief that insanity was always an affection of the reasoning or intellectual powers. On examining closely among the patients at the Bicetre, he found that there were many maniacs, who evinced no lesion whatever of the understanding, but were under the dominion of instinctive fury. The reality and importance of this distinct form of insanity is now recognized by several practical observers of the present day; they do not all, however, appear to concur with Pinel in thinking, that the integrity of the understanding is so fully preserved as he supposed. It has been observed that there is a great tendency in this affection to pass into the state of intellectual mania; some even go so far as to consider the former as belonging to the initiatory stage or incubation of the latter.

A very common feature of moral mania is a deep perversion of the social affections, whereby the feelings of kindness and attachment that flow from the relations of father, husband, and child, are replaced by a perpetual inclination to teaze and embitter the existence of others. This affection is far more common in the ordinary walks of society than is generally supposed. It is generally set down to eccentricity of temper, or inherently vicious dispositions. In such persons the suspicion of insanity is immediately dispelled by calling to mind the general correctness of their views, and the steadiness and sagacity with which they pursue their daily avocations. The consequences of these erroneous opinions are sometimes strikingly exhibited when a person thus afflicted becomes the object of a legal procedure. Whilst he may be described by one, as acute and methodical in his business, and rational in his discourse, and believed to be perfectly sane, another will testify to the strangest freaks that ever a madman played, and thence deduce the conviction of his insanity; while one represents him, as social and kindly in his disposition, ready to assist and oblige, and to accommodate himself to the varying humours of those about him, it will be testified by another that in his domestic relations, his former cheerfulness has given way to gloom and moroseness, that equanimity of temper has been replaced by frequent gusts of passion, and that the warm affections which spring from the relations of parent and child, husband and wife, have been transformed into indifference and hate. We shall now pass on to the legal consequences of mania.

### Legal Consequences of Intellectual Mania.

We have already seen that the common law relating to insanity is open to censure as well on account of the manner in which it modifies the civil and criminal responsibilities of the lunatic, as of the looseness, inconsistency, and incorrectness of the principles on which the fact of the existence of the disease is judicially established. The disabilities it imposes on the insane are founded no doubt on the most humane and enlightened views; it being certainly an act of the greatest mercy to incapacitate a person from making contracts, &c. who has lost his natural power of discerning and judging. These civil disabilities are not incurred however, by every one labouring under mental derangement; the measure of insanity necessary to produce this effect is a question reserved for judicial investigation, the result of which will no doubt depend on the views of individuals relative to the effect of insanity on the mental operations, and to the respect due to opinions and decisions already promulgated. General intellectual mania, or that form involving all or most of the operations of the understanding, should be attended to in the fullest extent, by the legal consequences of insanity; whilst partial intellectual insanity does not necessarily render a person non compos. With respect to the question, when mania invalidates a person's civil acts and annuls criminal responsibility, there has been much discussion, nor are there any general principles as yet established on the subject.

We know from experience that, notwithstanding the serious derangement of the reasoning power which a person must have experienced, who entertains the strange fancies that sometimes find their way into the mind, it may be exercised on all other subjects, so far as we can see, with no diminution of its natural soundness. To deprive such a person of the management of his affairs, or to invalidate his contracts, would be to inflict a certain and serious injury. The principle which our author would inculcate on this point is, "that monomania invalidates only when such act comes within the circle of the diseased operations of the mind." This principle would certainly be of easy application, if the insane delusion never varied; for then all we should have to do, would be to ascertain what the delusion is, and then determine whether the act in question comes within its influence. But unfortunately for the adequacy of this rule, the delusion is frequently changing, in which case it is not only difficult to determine how far it may have been connected with any particular act, but the mind, in respect to other operations, has lost its original soundness, to such an extent that it cannot be trusted in the management of important concerns. All we can do then, is, in doubtful instances, to be governed by the circumstances of the case, a course which seems far more rational than the practice of universal disqualification.

The author mentions one case, to which he thinks that his principles will not apply, and that is in determining the validity of a marriage contracted in a state of partial insanity. Here, he says, it is not sufficient to consider merely the connexion of the hallucination with the idea of being married, nor should he form any conclusion in favor of the capacity of the deranged party, from the propriety with which he conducts himself during the ceremony. In other contracts, all the conditions and circumstances may be definite and brought into view at once, and the capacity of the mind to comprehend them determined with comparative facility; whilst, on the contrary, in the contract of marriage, nothing is definite, the obligations which it imposes are of an abstract kind, and constantly varying. With respect to the principles that should regulate the legal relations of the partially insane, our author lays it down that, "whilst they should be left in possession of every civil right, that they are not clearly incapable of exercising, they should be subjected to the performance of no duties involving the interests or comfort of individuals, which may be equally well discharged by others." For this principle he states what we conceive a very sound reason, viz. that in the former instance we continue the enjoyment of a right that has never been abused; while in the latter, we refrain from imposing duties on persons not qualified to perform them.

In criminal, as well as in civil cases, it is important to consider the operation of the predominant idea, and its influence on the act in question, as there is no good reason why a person should be held responsible for a criminal act that springs from a delusion which would be sufficient to invalidate any civil act to which it might give rise, as a monomaniac's sense of the *fitness of things* is not different when he signs a ruinous contract, from what it is when he commits a criminal act. It is still a disputed point, whether partial mania should have the full legal effect of insanity, in criminal cases. Some writers will have it that the same principle which determines the effect of mania in civil, should also determine its effect in criminal cases; that is, that criminal responsibility should be annulled only when the act comes within the range of the diseased operations of the mind. In favor of this view, it may be urged that the connexion of the morbid delusion with the criminal act is generally very direct. Against this view it may be objected, that it is not always easy to trace the connexion between the predominant idea and the criminal act. The links which connect the thoughts that rise in succession in the sound mind, defy all our penetration, and the few laws we have established are totally inapplicable to the associations of the insane mind. Another objection to this view is, that the predominant idea is sometimes frequently changing, and at other times concealed by the patient.

Hoffbauer not only limits the exculpatory effects of partial insanity, to the acts which clearly come within its influence, but has laid down the principle, that in the criminal jurisprudence of this state, the predominant idea should be considered as true; that is, that the acts of the patient should be judged as if he had really been in the circumstances he imagined himself to be when they were committed. This is obviously based on the common, but erroneous maxim, that madmen reason correctly, but from wrong premises; we know, however, that there are numerous instances wherein the premises and conclusions are all equally erroneous. In the courts of justice in this country, there has been a great diversity of practice on this subject. Under the influence of Lord Hale's doctrines, partial insanity has seldom been considered as sufficient per se, to annul responsibility for crime; and when it has, it is generally in cases where the principal delusions were of a religious nature. We find that our analysis of this work has run out to so great a length that we must close it here; not, however, without expressing our decided approbation of the book. We recommend a careful perusal of it both to our medical and legal friends. The latter class of readers will see that there are certain points, even in the judicial discussions on insanity, where they would do well to consult something more defined than mere common sense.

